# Morphological and Molecular Characterization of *Meloidogyne arenaria* (Neal, 1889) Chitwood, 1949 Populations Parasitizing Pistachio in Kerman and Khorasan Razavi Provinces, Iran

**DOI:** 10.2478/jofnem-2024-0043

**Published:** 2024-10-28

**Authors:** Fatemeh Shekari Mahoonaki, Esmat Mahdikhani Moghadam, Mohammad Zakiaghl, Mohammad Moradi, Majid Pedram

**Affiliations:** Department of Plant Protection, Faculty of Agriculture, Ferdowsi University of Mashhad, Mashhad, Iran; Pistachio Research Center, Horticulture Sciences Research Institute, Agriculture Research Education and Extension Organization (AREEO), Rafsanjan, Iran; Department of Plant Pathology, Faculty of Agriculture, Tarbiat Modares University, Tehran, Iran

**Keywords:** *COII-16S*, *D2-D3*, morphology, morphometrics, *Nad5*

## Abstract

Root-knot nematodes (RKNs) are the most destructive nematode species in main pistachio cultivation areas of Iran, and adversely affect crop quality and yield. So far, *Meloidogyne incognita* and *M. javanica* have been reported infecting pistachio. In this study, four populations of *M. arenaria* were found infecting pistachio in Kerman and Khorasan Razavi provinces. The morphology and morphometrics of the recovered populations closely match the data given for other populations of the species. Molecular characterization of the recovered populations was performed by sequencing three genomic and mitochondrial regions, including D2-D3 of LSU rDNA, *COII-16S* and *Nad5* mtDNA. The D2-D3 sequences had more than 99% identity with many sequences of tropical species. The *COII-16S* sequences had more than 99% identity with sequences of *M. arenaria*, *M. morocciensis* and *M. thailandica*. The *Nad5* sequences had 99.81% identity with some sequences of *M. arenaria*. The D2-D3 and *COII-16S* sequences of *M. arenaria* did not form independent clades in corresponding Bayesian trees, but *Nad5* sequences formed a monophyletic group in corresponding phylogeny. Based on this study, *M. arenaria* is present in Iran’s pistachio gardens, necessitating appropriate management measures.

Pistachio is one of the most important economic agricultural products in the world and is well known for its nutritional and medicinal properties. It is most commonly cultivated in Kerman and Khorasan Razavi provinces in Iran (Ahmadi et al., 2016). Pistachio yield is reduced by different abiotic and biotic factors including root-knot nematodes (RKNs) (Fatemy, 2009; Fazeli Salmani et al., 2012). Annually, the estimated economic loss of plant-parasitic nematodes in the agricultural sector is nearly $125 billion (Mesa-Valle et al., 2020).

RKNs are the most economically important plant-parasitic nematodes on agricultural products in the world (Jones et al., 2013). The most common symptoms of RKNs infection on plants roots include galls, shoot chlorosis, stunted growth, nutrient deficiencies symptoms, and secondary infections (Subbotin et al., 2021). Fazeli Salmani et al. (2012) stated that RKN infection in pistachio causes the accumulation of nutrients such as potassium and zinc in the roots, causing the reduction of these nutrients in the branches and leaves, and this effect is well visible in the case of potassium, so that the plant shows the symptoms of potassium deficiency and leaf margin burning. Calcium deficiency has also been observed in infected trees. The symptoms of RKNs infection in pistachio gardens are generally in the form of spotting, reduced growth and stunted plants, reduced leaf number and surface, wilting in hot hours of the day, leaf margins burning, reduced crop production and root deformations and galls (Khatami Doost et al., 2017).

RKNs belong to the genus *Meloidogyne* Göldi, 1887, which has about 98 species (Subbotin et al., 2021). Effective management of RKNs is dependent on accurate species identification (Khanal et al., 2016). Identification of *Meloidogyne* species with traditional morphological methods is always challenging, due to the occurrence of mixtures of two or more species in the same field, morphological plasticity and overlapping morphometrics (Janssen et al., 2016). In order to solve this problem, molecular diagnosis using ribosomal and mitochondrial DNA sequences are recommended in addition to morphological information (Blok and Powers, 2009).

Several studies were carried out about RKNs in pistachio gardens in Iran. In a short report, Farivar Mehin (1986) reported three RKNs dominating pistachio gardens in Kerman province including *M. javanica* (Treub, 1885) Chitwood, 1949, *M. incognita* (Kofoid & White, 1919) Chitwood, 1949 and *M. arenaria* (Neal, 1889) Chitwood, 1949. Furthermore, *M. cruciani* (Garcia-Martinez, Taylor & Smart, 1982) was identified morphologically and reported from Kerman province (Shekari Mahoonaki et al., 2019). Following Farivar Mehin (1986), *M. javanica* and *M. incognita* were isolated and reported from pistachio gardens frequently, using morphological (Karegar, 1989; Hosseinipour, 1992; Madani et al., 2012) and both morphological and molecular tools (Zeynaddini Riseh, 2017). In the present survey, four populations of *Meloidogyne* spp. were recovered from pistachio gardens in Kerman and Khorasan Razavi provinces, Iran. All four recovered species were identified as *M. arenaria* using morphological and molecular tools. So far, *M. arenaria* populations infecting pistachio in Iran, were not characterized using integrative approaches. Therefore, the objective of the present report was the close study of the recovered populations of the species using this method.

## Materials and Methods

### Nematode populations

Several infested soil and root samples having *Meloidogyne* galls were collected from pistachio gardens in Iran (Rafsanjan and Nough in Kerman province; Feyzabad in Khorasan Razavi province) during 2018–2019. Samples were put into plastic bags and their data (assigned codes, locality, GPS information etc.) were tagged on each bag. The samples were then transferred to the laboratory for further processing. All RKN populations were reared on tomato (*Solanum lycopersicum* L. cv. Early Urbana) in a greenhouse.

### Morphological characterization

Egg masses and females were handpicked from infected tomato roots under a stereomicroscope. Second-stage juveniles (J2s) were hatched from egg masses incubated in 28°C, heat-killed by adding boiling 4% formaldehyde solution, transferred to anhydrous glycerin (De Grisse, 1969) and mounted on permanent slides. Perineal patterns were cut from preserved female specimens in lactic acid according to the method of Taylor and Netscher (1974) and were put on glycerin drop on a glass slide. Interphasmid distance, vulval slit length and distance from vulval slit to anus were measured using prepared perineal patterns. Anterior part of females which were cut in lactic acid, were used to measure stylet length, the distance from the stylet knobs to the dorsal pharyngeal gland orifice (DGO) and the excretory pore distance from anterior end. Fresh females in temporary slides were used to measure their body length and width. All specimens were observed using stereomicroscope and a light microscope (Olympus, BH-2). Interpretation of morphological features was based on the descriptions and illustrations given by Jepson (1987) and Subbotin et al. (2021). Information of the recovered populations of *M. arenaria* including the assigned codes, locality and GPS data are given in Table 1.

**Table 1.** Information of geographical distribution areas of *Meloidogyne* arenaria in two pistachio-growing provinces of Iran and newly generated sequences in present study.

**Population code**	**Sampling area**	**N**	**E**	**LSU D2-D3 GenBank accession No.**	***COII-16S* GenBank accession No.**	***Nad5* GenBank accession No.**
KN1-1	Kerman province, Nough	30′58′24.0″	55′35′35.8″	-	OR268637	OR264475
KR1-1	Kerman province, Rafsanjan	30′25′17.0″	55′49′40.9″	-	OR268638	OR264476
KhF1-2	Khorasan Razavi province, Feyzabad	34′58′36.8″	58′42′09.8″	OR267403	OR268639	-
KhF1-3	Khorasan Razavi province, Feyzabad	34′57′14.0″	58′39′28.1″	OR267404	OR268640	OR264477

### Molecular characterization

#### DNA extraction

A single female nematode specimen of each population was picked out and transferred to a small drop of TE buffer (10 mM Tris-Cl, 0.5 mM EDTA; pH 9.0, Qiagen) on a clean slide and squashed using a clean coverslip. The suspension was collected by adding 50 μl of TE buffer. DNA samples were stored at −20′C until used as PCR template.

#### PCR

The primers used for PCR and DNA sequencing are given in Table 2. The 25-μl mixture of PCR tubes contained 12.5-μl 2X *Taq* red master mix DNA polymerase (Amplicon, Stenhuggervej 22, DK-5230 Odense M, Denmark), 7.5-μl distilled water, 1-μl each of 10-μM forward and reverse primers and 3 μl of the DNA template. The PCR was performed in a Biometra^®^ thermocycler (Analytik Jena AG, Konrad-Zuse-Str. 1, 07745 Jena, Germany). The amplification of all loci was carried out under the following cycling conditions: 94′C for 5 min, then 35 PCR cycles of 94′C for 30 seconds, 55′C for 30 seconds, 72′C for 30 seconds and final incubation at 72′C for 10 min.

**Table 2.** List of primers used for sequencing of different loci in the present study.

**Locus**	**Primers**	**5′ to 3′ Sequence**	**Reference**
LSU rDNA D2-D3	D2Ab	ACAAGTACCGTGAGGGAAAGTTG	De Ley et al. (1999)
	D3B	TCGGAAGGAACCAGCTACTA	
*COII-16S*	C2F3	GGTCAATGTTCAGAAATTTGTGG	Powers and Harris (1993)
	1108	TACCTTTGACCAATCACGCT	
*NADH dehydrogenase subunit 5* (*Nad5*)	NAD5F	TATTTTTTGTTTGAGATATATTAG	Janssen et al. (2016)
	NAD5R	CGTGAATCTTGATTTTCCATTTTT	

#### Observing PCR results

Amplified PCR products were loaded into 1% agarose gel and were electrophoresed at 80 V for 40 min using Green-Viewer® for staining the gel.

#### Sequencing and molecular profiles

The successfully amplified PCR products were sequenced using the same primers used in PCR by Macrogen Co., South Korea. The accession numbers of newly obtained sequences are given in Table 1.

Basic local alignment search tool for nucleotide (BLASTn) at the National Center for Biotechnology Information (NCBI) was used to unravel the identity of the new sequences compared with previously deposited sequences. The relevant sequences of each locus were downloaded in order to infer the phylogenetic relationships of the newly generated sequences. The D2-D3 expansion fragment of LSU rDNA of *Pratylenchus penetrans* (Cobb, 1917) Filipjev & Schuurmans Stekhoven, 1941, the mitochondrial *COII-16S* sequence of *Radopholus similis* (Cobb, 1893) Thorne, 1949 and *Pratylenchus vulnus* Allen & Jensen, 1951, and *Nad5* sequences of two isolates of *M. enterolobii* Yang & Eisenback, 1983 (MT683468 and MG948240) were selected as outgroups for each corresponding phylogeny.

The Q-INS-i algorithm of the online version of MAFFT (version 0.91b) (https://mafft.cbrc.jp/alignment/server/) (Katoh and Standley, 2013) was used to align three datasets and the resultant alignment was manually edited using MEGA7 (Kumar et al., 2016). The model of base substitution was selected using MrModeltest 2 (Nylander, 2004). The Akaike-supported model, a general time reversible model, including among-site rate heterogeneity and estimates of invariant sites (GTR+G+I) was used in phylogenetic analyses of three loci. Bayesian analyses were performed using MrBayes v3.1.2 (Ronquist and Huelsenbeck, 2003) running the chains for 2 million generations (for the three datasets). After discarding burn-in samples (the burn-in was set to 25%), the remaining samples were retained for further analyses. The Markov chain Monte Carlo (MCMC) method within a Bayesian framework was used to estimate the Bayesian posterior probabilities (BPPs) of the phylogenetic trees (Larget and Simon, 1999) using the 50% majority rule. Convergence of model parameters and topology were assessed based on average standard deviation of split frequencies and potential scale reduction factor values. Adequacy of the posterior sample size was evaluated using autocorrelation statistics as implemented in Tracer v.1.7 (Rambaut et al., 2018). The output files of the trees were visualized using Dendroscope v3.2.8 (Huson and Scornavacca, 2012) and were digitally drawn in CorelDRAW software version 2020.

## Results

### RKN identification

**Figure 1:**
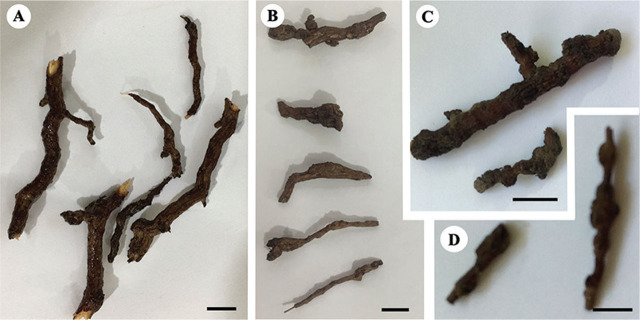
Photographs of root galls caused by *Meloidogyne arenaria* on pistachio in Kerman and Khorasan Razavi provinces, Iran. A: Irregular galls by isolate KR1-1; B: Irregular galls by KN1-1 isolate; C: Irregular galls by KhF1-2 isolate; D: Spheroid galls by KhF1-3 isolate. (All scale bars = 10 mm).

The four recovered populations of *Meloidogyne* (codes KR1-1 and KN1-1 from Kerman province; and codes KhF1-2 and KhF1-3 from Khorasan Razavi province) causing galls on pistachio roots (Fig. 1) were identified as *M. arenaria* based on the combined analyses of morphological and molecular data.

### Pathogenic symptoms

The infected roots of host pistachio trees and the inoculated tomato plants roots in the greenhouse had large and compound galls which contained two or more females. The bodies of females were completely or mostly embedded inside gall tissue. The shape of galls created by population KhF1-3 in the greenhouse on the tomato were spheroid, but those created by population KR1-1, KN1-1 and KhF1-2 were irregular.

### Morphological profile

#### Females (based on four recovered populations)

(Figs. 2, 3).

**Figure 2:**
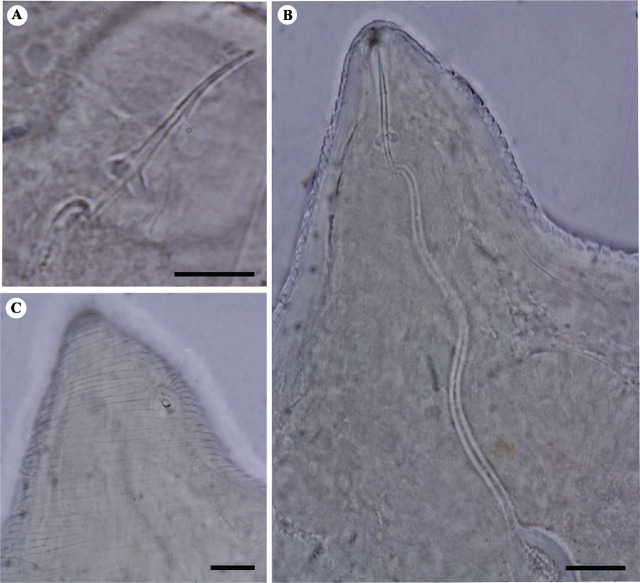
Light microphotographs of stylet and cephalic region of Iranian population of *M. arenaria* (isolate KhF1-2). A: Stylet, B: Anterior body region, C: The excretory pore. (All scale bars = 10 μm).

**Figure 3:**
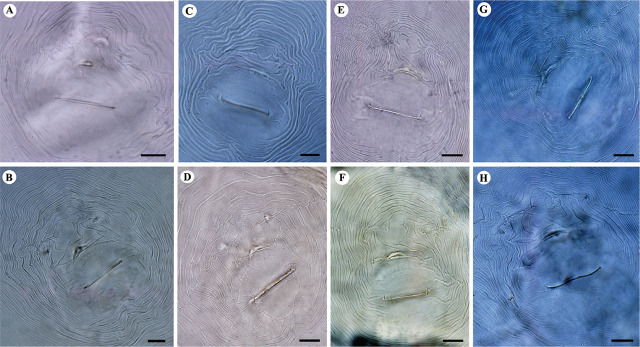
Light microphotographs of perineal patterns of studied *M. arenaria* populations. A, B: Isolate KR1-1; C, D: Isolate KN1-1; E, F: Isolate KhF1-2; G, H: Isolate KhF1-3; A, C, E, G: The dorsal arch is high; B, D, F, H: The dorsal arch is moderately high, phasmids are distinct. (All scale bars = 10 μm).

For morphometrics, see Table 3.

**Table 3.** Morphometrics^a^ of females of four *Meloidogyne arenaria* populations from pistachio gardens in Iran compared with other populations. Measurements in μm.

	**This study**					**Previous studies**

**Character**	**KR1-1**	**KN1-1**	**KhF1-2**	**KhF1-3**	**Total**	

**n**	**7**	**7**	**7**	**7**	**28**	
Body length	804.3±62.9 (720–890)	849.3±46.0 (770–900)	862.9±24.3 (840–900)	875±44.3 (810–940)	847.9±51.6 (720–940)	741±115^b^ (601–985)
Body width	542.9±46.8 (480–590)	628.6±27.3 (590–670)	578.6±27.9 (550–620)	649.3±31.7 (590–685)	599.8±53.4 (480–685)	448±89^b^ (334–626)
Stylet	16.6±0.6 (15.5–17.0)	17.1±0.2 (17.0–17.5)	16.6±0.5 (16–17)	17.4±0.6 (17.0–18.5)	16.9±0.6 (15.5–18.5)	15.1±0.05^c^ (13–17) n=150
DGO	4.1±0.4 (3.5–4.5)	5.4±0.7 (4–6)	4.4±0.5 (4–5)	4.9±0.4 (4.5–5.5)	4.7±0.7 (3.5–6)	4.8±0.06^c^ (3.1–6.6) n=150
Excretory pore to anterior end	41.0±5.4 (35–49)	41.9±4.0 (39–50)	36.3±3.9 (31–42)	30.4±3.0 (25–34)	37.4±6.1 (25–50)	42.2±0.94^c^ (18–80) n=150
Vulval slit	23.1±1.6 (21–25)	27.9±2.6 (25–33)	21.2±1.8 (19–24)	25.4±1.1 (24–27)	24.4±3.1 (19–33)	29.3±4.1 (24–37) n=9^b^
Vulva-anus	16.6±1.4 (15–19)	19.6±1.2 (18–21)	19.1±2.0 (16.5–22.0)	20.4±0.9 (19.0–21.5)	18.9±1.9 (15–22)	20.7±2.6 (18–21) n=9^b^
Interphasmid distance	27.9±2.0 (25–31)	35.0±8.3 (22–47)	28.9±2.8 (25–33)	33.3±3.5 (27–38)	31.3±5.4 (22–47)	28–31^d^

a Mean ± standard deviation (range).

b Jepson (1987).

c Cliff and Hirschmann (1985).

d Chitwood (1949).

Body pear-shaped, no posterior terminal protuberance, 720–940 μm long. Stylet 15.5–18.5 μm, conus curved dorsally, gradually tapering to blunt tip anteriorly, its shaft broad, cylindrical, gradually widening posteriorly and basal knobs rounded to teardrop shaped. Excretory pore posterior to stylet knobs (distance from anterior end in resting position of stylet, 25–50 μm). Perineal pattern variable, rounded to ovoid with fine to coarse striae. Dorsal arch moderately high to high, with striae smooth or slightly wavy, continuous or broken, slightly bent towards tail tip at lateral line; generally forming shoulders on lateral portion of arch. Lateral field distinct, slightly irregular. Vulval slit narrow, 19–33 μm long.

#### Male

*Not common.* The only one male specimen found in KhF1-2 population, was lost during processing.

#### Second-stage juveniles

(Figs. 4, 5).

**Figure 4:**
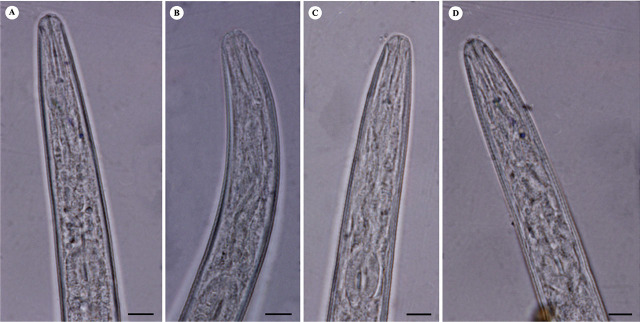
Light microphotographs of anterior portion of second-stage juveniles of studied *M. arenaria* populations. A: Isolate KR1-1; B: Isolate KN1-1; C: Isolate KhF1-2; D: Isolate KhF1-3. (All scale bars = 5 μm).

**Figure 5:**
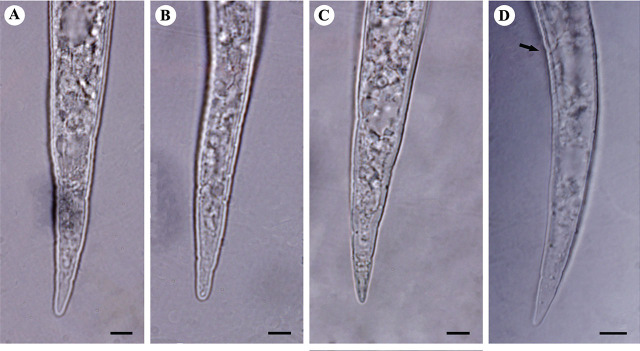
Light microphotographs of tail of second-stage juveniles of studied *M. arenaria*. populations. A: Isolate KR1-1; B: Isolate KN1-1; C: Isolate KhF1-2; D: Isolate KhF1-3, arrow shows anus. (All scale bars = 5 μm).

For morphometrics, see Table 4.

**Table 4.** Morphometrics^a^ of second-stage juveniles of four populations of *Meloidogyne arenaria* from pistachio gardens in Iran and other populations. Measurements in μm.

	**This study**					**Previous studies**

**Character**	**KR1-1**	**KN1-1**	**KhF1-2**	**KhF1-3**	**Total**	

**n**	**7**	**6**	**6**	**7**	**26**	
Body length	464±48 (378–543)	458±59 (393–537)	355±24 (335–400)	419±29 (390–460)	425±58 (335–543)	504±4.3^b^ (392–605)
Body width	16.3±2.1 (13.5–19.0)	14.2±0.6 (13.5–15.0)	16.6±2.3 (14.5–20.0)	15.1±1.1 (14–17)	15.5±1.8 (13.5–20.0)	15.3±0.1^b^ (13–18)
Stylet	11.0±0.7 (10–12)	11.3±0.5 (10.5–12.0)	12.0±0.9 (11–13)	11.4±0.8 (10.5–12.5)	11.4±0.8 (10–13)	11.1±0.03^b^ (10–12)
DGO	3.5±0.6 (3.0–4.5)	3.2±0.3 (3.0–3.5)	3.3±0.4 (3–4)	3.1±0.2 (3.0–3.5)	3.3±0.4 (3.0–4.5)	3.7±0.04^b^ (3–5)
Median bulb	57±7.2 (52.5–73.0)	54±1.8 (52–57)	52.1±3.7 (48.5–58.0)	56.1±3.1 (52.5–60.0)	55.2±4.7 (48.5–73.0)	60.9±0.43^b^ (49.4–71.2)
Excretory pore to anterior end	74.9±8.6 (56.5–80.0)	77.1±9.2 (65–90)	81.4±6.9 (75–92)	77.9±6.9 (64–85)	77.7±7.8 (56.5–92.0)	89.8±0.56^b^ (75.0–105.2)
Tail length	47.9±4.8 (41–53)	46.6±4.7 (38–51)	42.2±6.2 (36.5–54.0)	47.9±3.2 (45–54)	46.3±5.1 (36.5–54.0)	56.0±0.43^b^ (44–59)
Hyaline	16.6±2.7 (13–20)	16.3±2.2 (14.0–19.5)	13.3±1.5 (12–16)	14.9±1.0 (13.5–16.0)	15.3±2.3 (12–20)	14.0±3.3^c^ (10–21) n=17

a Mean ± standard deviation (range)

b Cliff and Hirschmann (1985).

c Skantar et al. (2008).

Body slender, tapering at both ends, 335–543 μm long. Labial region rounded and continuous with adjacent body. Lateral field with four incisures. Stylet slender, 10–13 μm long, knobs rounded and small. Tail conoid with rounded terminus, 36.5–54.0 μm long. Hyaline region not well differentiated, 13–20 μm long.

#### Remarks

The polytomous identification codes of Iranian populations of *M. arenaria* according to Subbotin et al. (2021) are as follows: *Female*: A21, B21, C2, D4; *J2*: A231, B32, C3, D21, E2, F3. The body length and width of females of four populations ranged from 720 to 940 μm and 480 to 685 μm, respectively. The isolates KR1-1 had the shortest body and KhF1-3 had the longest body, and similarly, the lowest and highest body width. Stylet length showed variation among the four populations, which ranged from 15.5 to 18.5 μm. Isolate KR1-1 had the shortest stylet and isolate KhF1-3 had the longest stylet. The dorsal pharyngeal gland orifice (DGO) distance from the anterior end ranged from 3.5 to 6 μm. The shortest DGO belonged to isolate KR1-1 and the longest belonged to KN1-1. The excretory pore distance from the anterior end ranged from 25 to 50 μm, belonging to isolate KhF1-3 and KN1-1. The ratio of excretory pore distance from the anterior end to stylet length in KR1-1, KN1-1, KhF1-2 and KhF1-3 were 2.5, 2.5, 2.2 and 1.7, respectively. Perineal patterns were slightly variable between populations/individuals, but the common shapes were shown in Fig. 3. The dorsal arch in all populations was moderately high to high. The striae in all populations were coarse and, in the arch, indented at lateral lines, forming a shoulder. Some specimens in all four populations showed wings. The vulval slit ranged from 19 to 33 μm, the shortest was in isolate KhF1-2 and the longest was in isolate KN1-1. The distance between anus to vulval slit ranged from 15 to 22 μm, the shortest was in isolate KR1-1 and the longest was in isolate KhF1-2. The interphasmid distance ranged from 22 to 47 μm, the shortest and the longest were both in specimens of the isolate KN1-1.

The body length of J2s ranged from 335 to 543 μm, the shortest was in isolate KhF1-2 and the longest was in isolate KR1-1. Their body width ranged from 13.5 to 20.0 μm, belonging to isolate KN1-1 and isolate KhF1-2, respectively. Their stylet length ranged from 10 to 13 μm in isolate KR1-1 and isolate KhF1-2 Their DGO length ranged from 3.0 to 4.5 μm, the longest belonging to isolate KR1-1. The anterior end to median bulb valve distance ranged from 48.5 to 73 μm, belonging to KhF1-2 and KR1-1, respectively. Excretory pore distance from anterior end ranged from 56.5 to 92 μm in isolate KR1-1 and KhF1-2. Tail length ranged from 36.5 to 54 μm in isolate KhF1-2 and KhF1-3. The hyaline region of tail ranged from 12 to 20 μm in isolates KhF1-2 and KR1-1, respectively. The common form of tail in all populations is given in Fig. 5.

### Molecular analyses

#### BLAST search

**Figure 6:**
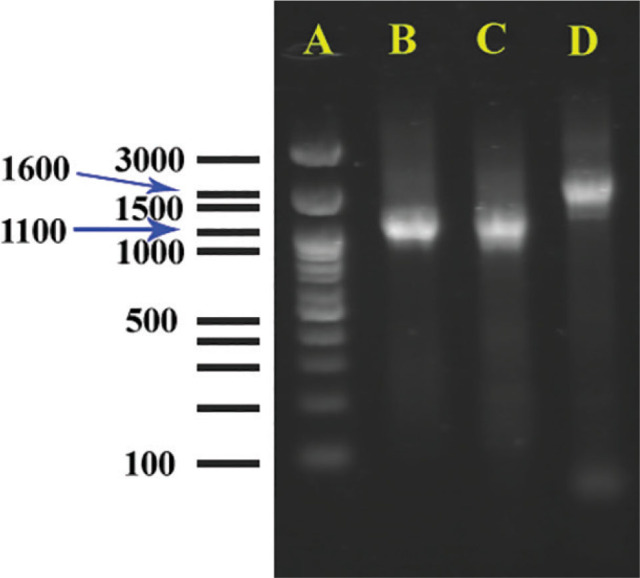
Amplification products generated from individual females with mitochondrial primer sets C2F3/1108. A) Ladder. B, C) Unique 1100-bp product if *Meloidogyne arenaria*, B isolate KhF1-2, C isolate KhF1-3, D) Unique about 1600-bp product of *Meloidogyne javanica*.

The size of PCR products of *COII-16S* sequence of all studied populations were about 1.1 kb. Amplification products generated from individual females with mitochondrial primers C2F3/1108 for isolates KhF1-2 and KhF1-3 are shown in Fig. 6 B-C. Fig. 6D shows amplification product of *COII-16S* of *M. javanica* that is about 1.6 kb, for comparison purposes.

The two D2-D3 sequences of two isolates KhF1-2 and KhF1-3 were identical while aligning. BLAST search using these sequences revealed they have 99% identity with D2-D3 sequences of several tropical species including *M. incognita*, *M. javanica*, *M. arenaria, M. konaensis, M. morocciensis, M. luci, M. haplanaria, M. paranaensis, M. phaseoli, M. lopezi, M. arabicida, M. izalcoensis* and *M. floridensis* formerly deposited into GenBank database.

The four newly generated *COII-16S* sequences were almost identical while aligning and had 99% identity with *COII-16S* sequences of *M. arenaria, M. morocciensis* and *M. thailandica* formerly deposited into GenBank database.

The three newly generated sequences of *Nad5* were identical while aligning and had 99.81% identity with same other sequences of different populations of *M. arenaria* formerly deposited into GenBank database.

### Molecular phylogenetic relationships

**Figure 7:**
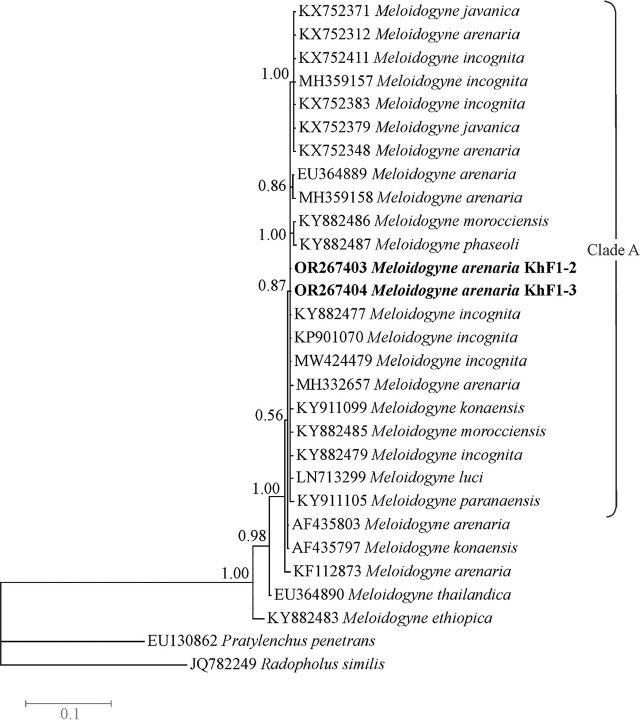
Bayesian 50% majority rule consensus tree inferred from LSU rDNA D2-D3 segment of *Meloidogyne arenaria* populations recovered from pistachio gardens of Iran under the GTR + G + I model. Bayesian posterior probabilities (BPP) more than 50% are given for appropriate clades. Newly generate sequences are in bold font.

The LSU dataset was composed of 29 sequences of species/isolates with two outgroup sequences of *Pratylenchus penetrans* and *Radopholus similis*. The phylogenetic tree based on this dataset is presented in Fig. 7. In this tree, the LSU sequences of two isolates KhF1-2 and KhF1-3 have placed in a monophyletic polytomous clade (clade A) that also includes sequences of other tropical RKN species including *M. incognita, M. arenaria*, *M. konaensis*, *M. morocciensis*, *M. luci* and *M. paranaensis.* This clade received 0.87 BPP. Two sequences assigned to *M. arenaria* (AF435803, KF112873) have placed out of the clade A.

**Figure 8:**
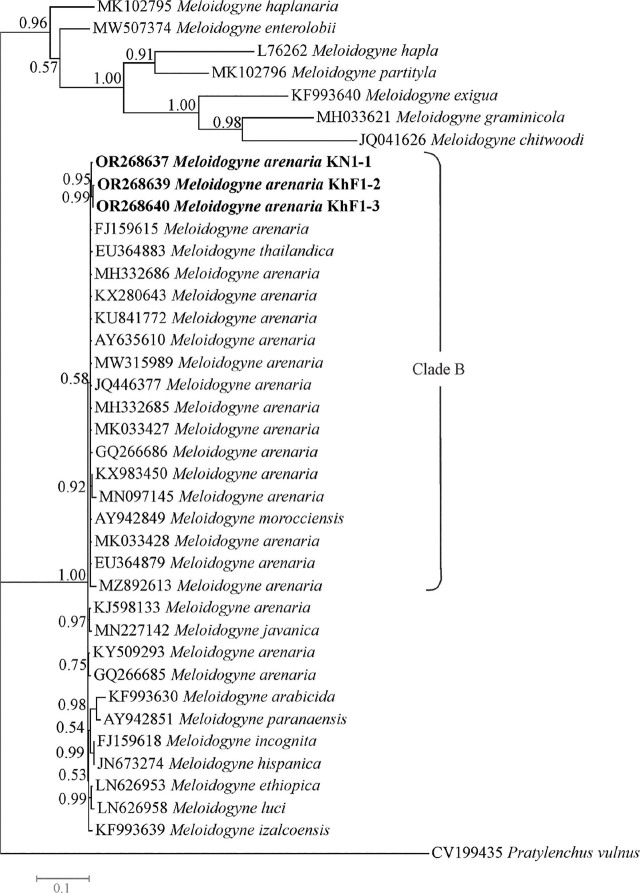
Bayesian 50% majority rule consensus tree inferred from mitochondrial *COII-16S* segment of *Meloidogyne arenaria* populations recovered from pistachio gardens of Iran under the GTR + G + I model. Bayesian posterior probabilities (BPP) more than 50% are given for appropriate clades. Newly generated sequences are in bold font.

The *COII-16S* dataset was composed of 39 sequences of species/isolates with one sequence of *Pratylenchus vulnus* as outgroup. The phylogenetic tree based on this dataset is presented in Fig. 8. The *COII-16S* sequence of the population KR1-1 was not included in this tree because of its short length. In this tree, three newly generated sequences of Iranian populations of *M. arenaria* occupied placements inside a major clade (the clade B) that also includes several sequences of *M. arenaria*, *M. morocciensis* and *M. thailandica.* This clade was poorly supported (0.58 BPP).

**Figure 9:**
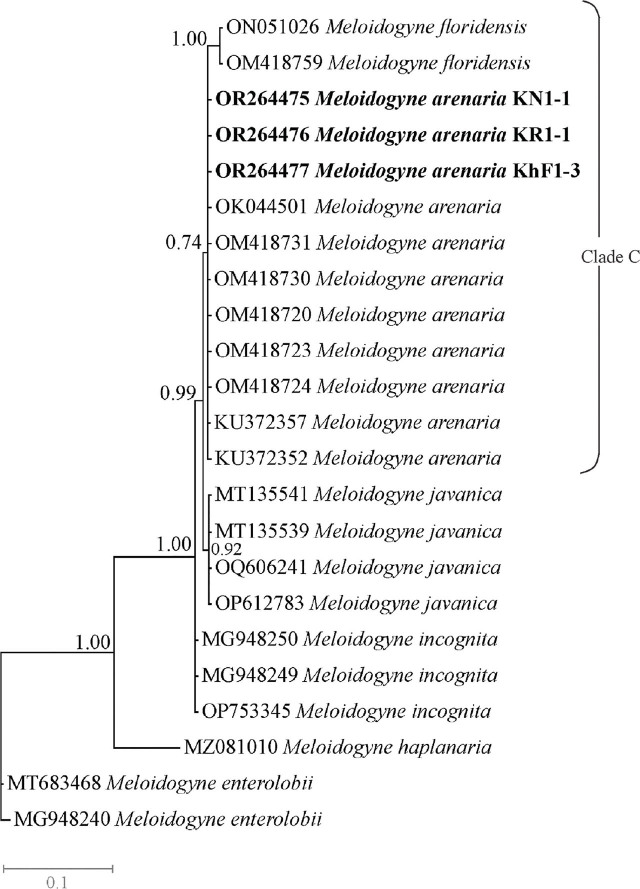
Bayesian 50% majority rule consensus tree inferred from mitochondrial *Nad5* segment of *Meloidogyne arenaria* recovered from pistachio gardens of Iran under the GTR + G + I model. Bayesian posterior probabilities (BPP) more than 50% are given for appropriate clades. Newly generated sequences are in bold font.

The *Nad5* dataset was composed of 23 sequences of species/isolates with two outgroup sequences belonging to *M. enterolobii* (MT683468 and MG948240). The phylogenetic tree based on this dataset is presented in Fig. 9. In this tree, sequences of *M. arenaria* (the newly generated sequences plus sequences previously deposited in the database) and *M. floridensis* have formed a clade with 0.74 BPP (the clade C), and sequences of *M. floridensis* have appeared as a distinct lineage.

## Discussion

Pistachio is an important economic crop in Iran and the world. In this study, four populations of *M. arenaria* recovered from pistachio gardens of Kerman and Khorasan Razavi provinces were studied using morphological, morphometric and molecular data. *M. arenaria* was reported from Iran for the first time by Farivar Mehin (1986) on pistachio in Kerman province. However, no morphometric and molecular data of Iranian populations were provided to date. In a recent study, morphological and molecular data of a Vietnamese population of *M. arenaria* parasitizing black pepper have been given by Le et al. (2023).

All four recovered populations of *M. arenaria* produced large and compound irregular galls which contained two or more females on tomato plants in greenhouse cultures on tomato. The bodies of females were completely or mostly embedded inside gall tissue. According to Jepson (1987), *M. hapla, M. incognita, M. wartellei, M. grahami, M. thamesi, M. arenaria* and *M. javanica,* induce large and compound galls with deeply embedded females. Different symptoms may also occur on different host plants and as a result, species-typical symptoms could not be used for *Meloidogyne* species delimitation.

Differences in polytomous identification codes of Iranian populations of *M. arenaria* were observed in comparison with those given by Subbotin et al. (2021) as follow *Female*: A21, B21, C2, D4 vs A213, B2, C2, D4; *J2*: A231, B32, C3, D21, E2, F3 vs A2, B3, C213, D324, E3, F2. Some morphometrics e.g. female body length and body width of four recovered populations were in accordance with the data given for *M. arenaria* (Jepson, 1987) and *M. morocciensis* (Rammah and Hirschmann, 1990), however, their mean values were mostly in accordance with those given for *M. arenaria* race 1 reported from Florida (Osman et al., 1985). The stylet length of all populations was congruent with the ranges given for *M. arenaria* by Cliff and Hirschmann (1985) and *M. morocciensis* (Rammah and Hirschmann, 1990), and again, their mean value was mostly in accordance with the data given for *M. morocciensis*. The stylet length, however, is higher than that reported for *M. floridensis* and *M. thailandica*. The DGO mean values were similar to those of *M. arenaria* (Cliff and Hirschmann, 1985). The excretory pore to anterior end mean values were mostly close to those given for *M. arenaria* and shorter than that for *M. morocciensis*. Despite overlapped data between two species, *M. arenaria* and *M. morocciensis*, the ratio of excretory pore distance from anterior end to stylet length seems differs (Subbotin et al., 2021). This ratio is lower in *M. arenaria* (2.0–3.4 vs 3.5–5.0 in *M. morocciensis*). Vulval slit and vulva to anus distance were similar to the ranges given for *M. arenaria* and *M. morocciensis*, but interphasmid distances were similar to the ranges given for *M. arenaria* (22–47, after Chitwood, 1949). Variation in the perineal patterns of four studied populations were observed, but the wing-like pattern common for *M. arenaria* was observed. This pattern was not described for *M. morocciensis* (Subbotin et al., 2021). The dorsal arch in all populations were moderately high to high compared with the pattern illustrated for *M. arenaria*, and resembling that in *M. morocciensis*, but Cliff and Hirschmann (1985) showed a remarkable variation in perineal pattern of *M. arenaria*, similar to those observed in presently studied populations.

Males were uncommon in currently studied populations, which aligns with Jepson (1987), who argues that males may be rare in facultative and obligatory parthenogenetic species especially in field samples, and it may be necessary to induce their generation in culture.

Contrary to the female body length that was almost consistent among populations, the J2 body length was variable. The body length of the J2 of isolates KR1-1, KN1-1 and KhF1-3 were 335–543 μm long, resembling those given for race 2 of *M. arenaria* common in Florida (Osman et al., 1985) and the data given by Hirschmann and Rammah (1993). They were, however, similar to the ranges given for *M. morocciensis* by Rammah and Hirschmann (1990). The isolate KhF1-2 had the least J2 body length, lower than the data given for *M. arenaria* and *M. morocciensis* (Rammah and Hirschmann, 1990), but fitting the ranges given for the Vietnamese population of *M. arenaria* (Le et al., 2023) and those of *M. morocciensis* from Southern Brazil (Silva et al., 2020) infecting peach. The J2 stylet length in KR1-1, KN1-1 and KhF1-3 isolates was similar to those of *M. arenaria*; however, in KhF1-2, it was similar to that of *M. arenaria* (Osman et al., 1985) and *M. morocciensis* but higher than the ranges of *M. floridensis* and *M. thailandica*. The ranges of DGO, median bulb and excretory pore positions were similar in studied populations, close to the ranges given for *M. arenaria* and *M. morocciensis*. Tail length of J2 in all populations were shorter than those described for *M. arenaria* and *M. morocciensis* (Rammah and Hirschmann, 1990) and *M. thailandica*, but it is similar to the range of Vietnamese populations of *M. arenaria* Silva et al., 2020, *M. morocciensis* from Southern Brazil (Silva et al., 2020), and *M. floridensis* (Handoo et al., 2004). The hyaline region of tail of KhF1-2 and KhF1-3 isolates were similar, resembling that of *M. arenaria* and *M. morocciensis*, but it was higher in KR1-1 and KN1-1 isolates.

The result of LSU phylogeny was similar to previous studies (Kiewnick et al., 2014; Trinh et al., 2022; Le et al., 2023), corroborating it does not yield on a reliable resolution, which is helpful for delimiting tropical RKNs (*M. incognita-*group species).

As already stated, C2F3/1108 primers amplified a segment about 1.1 kb for the studied populations. Powers and Harris (1993) stated that C2F3/1108 primers amplify products of different lengths for RKN species. The result of the present *COII-16S* phylogeny was similar to previous studies (Tigano et al., 2005; Onkendi and Moleleki 2013; Ye et al., 2021; Murata et al., 2023).

The *Nad5* locus has proven to be a reliable marker for RKNs diagnostics, capable in identification of Incognita group species including *M. incognita*, *M. javanica, M. arenaria* and *M. floridensis* (Janssen et al., 2016). Similar to the resolved topology by Riascos et al. (2019) and Le et al. (2023), *M. arenaria* has separated from other tropical species in the corresponding tree and *M. floridensis* appeared as an independent lineage.

## Conclusion

The four presently recovered/studied populations of root knot nematode were identified as *M. arenaria* using an integrative approach.

On the base of this study, *M. arenaria* is developing at least in some pistachio gardens of the Kerman and Khorasan Razavi provinces and adopting appropriate strategies is necessary for its management.
